# Human Endometrial Side Population Cells Exhibit Genotypic, Phenotypic and Functional Features of Somatic Stem Cells

**DOI:** 10.1371/journal.pone.0010964

**Published:** 2010-06-24

**Authors:** Irene Cervelló, Claudia Gil-Sanchis, Aymara Mas, Francisco Delgado-Rosas, José Antonio Martínez-Conejero, Amparo Galán, Alicia Martínez-Romero, Sebastian Martínez, Ismael Navarro, Jaime Ferro, José Antonio Horcajadas, Francisco José Esteban, José Enrique O'Connor, Antonio Pellicer, Carlos Simón

**Affiliations:** 1 Fundación IVI-Instituto Universitario IVI, Universidad de Valencia, Valencia, Spain; 2 Valencia Stem Cell Bank, CIPF, Valencia, Spain; 3 iGenomix, Valencia, Spain; 4 Laboratory of Cytomics, CIPF, Valencia, Spain; 5 Department of Experimental Biology, Systems Biology Unit, University of Jaén, Jaén, Spain; 6 Department of Obstetrics and Gynecology, Hospital Universitario “La Fe”, Valencia, Spain; Brunel University, United Kingdom

## Abstract

During reproductive life, the human endometrium undergoes around 480 cycles of growth, breakdown and regeneration should pregnancy not be achieved. This outstanding regenerative capacity is the basis for women's cycling and its dysfunction may be involved in the etiology of pathological disorders. Therefore, the human endometrial tissue must rely on a remarkable endometrial somatic stem cells (SSC) population. Here we explore the hypothesis that human endometrial side population (SP) cells correspond to somatic stem cells. We isolated, identified and characterized the SP corresponding to the stromal and epithelial compartments using endometrial SP genes signature, immunophenotyping and characteristic telomerase pattern. We analyzed the clonogenic activity of SP cells under hypoxic conditions and the differentiation capacity *in vitro* to adipogenic and osteogenic lineages. Finally, we demonstrated the functional capability of endometrial SP to develop human endometrium after subcutaneous injection in NOD-SCID mice. Briefly, SP cells of human endometrium from epithelial and stromal compartments display genotypic, phenotypic and functional features of SSC.

## Introduction

The uterus is a major female reproductive organ lined by the human endometrium. It is a hormone-dependent, cyclically regenerating tissue that undergoes phases of growth, differentiation, decidualization and breakdown during menstruation. Within 5 to 6 days after the onset of menstruation, endometrial glands and stroma regenerate from their remnants in the basalis during the proliferative phase to form a new functionalis layer of 5–7 mm [1], [2]. This remarkable tissue remodeling capability occurs approximately 500 times during a woman's reproductive life-time, and also in the postmenopause with proper hormone replacement therapy.

Somatic stem cells (SSC) are defined as a restricted subpopulation of quiescent, slow-cycling, undifferentiated resident cells, characterized by a high proliferative capacity, multipotentiality, capability of self-renewal and the ability to form the tissue that they originate from. The SSC population has been identified in many adult tissues such as the central nervous system [3], [4], small intestine [5], [6], skin [7], bone marrow [8]–[10], retina [11] and skeletal muscle [12], [13].

The existence of endometrial SSC has been postulated, and different groups have demonstrated the existence of SSC-like cells in the human [14]–[21] and murine endometrium [22]–[23] using different approaches.

The Side Population (SP) method is based on the capability of SP cells to extrude the DNA binding dye Hoechst 33342 via the ATP-binding cassette [24]–[26]. ABC transporters, which include p-glycoprotein and ABCB1, can be specifically blocked by verapamil [27]. This method has been used for the identification of putative SSC in skin [28], myometrium [29], lung [30] and dental pulp [31]. Besides, this method has also been proposed, but not functionally demonstrated in vivo, in the human endometrium [32], [21].

Here we report that, SP cells from the stromal and/or the epithelial compartments in the human endometrium display genotypic, immunophenotypic, telomerase activity pattern and functional characteristics of endometrial stem cells.

## Methods

### Human tissue collection

This study was approved by the Instituto Universitario-IVI Institutional Review Board and Ethics Committee (Universidad de Valencia, Spain), and by the Ethics Committee of the Hospital Universitario Dr. Peset (Valencia, Spain). A written informed consent was obtained from each patient before tissue collection.

A total of 128 human endometrial biopsies using a Pipelle catheter (Genetics, Namont-Achel, Belgium) under sterile conditions, and 10 endometrial samples from uteri collected after hysterectomies performed for non-malignant indications in healthy women (from 18 to 48 years) throughout the menstrual cycle.

### Epithelial and stromal separation

Epithelial and stromal fractions were isolated using an established protocol [33] with modifications. Briefly, samples were carefully dissected and minced into 1–2 mm^3^ fragments, and enzymatically digested in DMEM (Sigma-Aldrich, Spain) containing 10 mg/ml collagenase type IA (Sigma-Aldrich, Spain). Stromal cells (single cells or small aggregates) and epithelial glands were separated on a size basis using gravity sedimentation and membrane filtration.

Cell suspensions were treated with erythrocyte lysis solution and evaluation of cell viability was performed with Propidium Iodide (PI; 5 µg/ml (Sigma-Aldrich, Spain)).

To confirm the purity of the fractions obtained, suspensions were stained with a typical epithelial antibody CD9-FITC (10 µl; Chemicon International; Anti-CD9, Clone MM2/57 FITC Conjugated, CBL162F) and stromal antibody CD13-PE (10 µl; Abcam, FACS - CD13 antibody [B-F10] Phycoerythrin, ab46883). Cells were analyzed in a Cytomics FC500 flow cytometer (Beckman-Coulter, CA, USA).

### Flow cytometric analysis

A 530/40 band-pass filter was used for fluoresceinisothiocyanate (FITC)-conjugated antibodies, while a 580/30 band-pass filter was employed for phycoeritrin (PE)-conjugated antibodies. To discriminate dead cells, a PI dot plot was used for FITC labeling. For the PE antibodies, 7-aminoactinomycin D (7-AAD) (Invitrogen, Spain) was detected using a 630/30 band-pass filter, and was used to exclude dead cells.

### Hoechst 33342 labeling

Cells isolated from the endometrial epithelial and stromal fractions were resuspended in DMEM pre-warmed at 37°C and supplemented with 2% FBS (Gibco, Spain) and 10 mM HEPES (Sigma-Aldrich, Spain). The cell suspension was labeled in the same media with 5 µg/ml of Hoechst 33342 dye (Ho-33342) (Sigma-Aldrich, Spain), either alone or in combination with 100 µM verapamil (Vp) (Sigma-Aldrich, Spain) [26], in a water bath at 37°C for 90–120 minutes. Then, cells were centrifuged for 6 minutes at 4°C, and were resuspended in cold HBSS supplemented with 2%FBS and 10 mM HEPES. PI permits to exclude dead cells prior to the flow cytometric analysis and sorting.

### Isolation of human endometrial SP cells

Cells were analyzed and sorted by a MoFlo® (Dako, Denmark, http://www.dako.com) jet-in-air high speed sorter. Excitation was performed with a water cooled Enterprise II ion laser (Coherent, CA, USA) which operated at the 351 nm and 488 nm wavelengths, and worked at 30 mW. Hoechst 33342 blue and red fluorescences were detected through 405/30 and 670/20 nm band-pass filters respectively, by measuring the signals on a linear scale. PI fluorescence was detected through a band-pass filter of 613/20 on a logarithmic scale. The gates for cell sorting were defined to collect live cells with a low Hoechst 33342 fluorescence (SP fraction), as well as live cells with a high Hoechst 33342 fluorescence (NSP fraction).

### RNA Isolation

Total RNA was extracted using the ‘TRIzol method’ according to the protocol recommended by the manufacturer (Life Technologies Inc., Gaithersburg, MD).

### Microarrays hybridization

Endometrial tissue consisted in a total of 8 endometrial biopsies (epithelial (n = 8) and stromal (n = 8) endometrial cell suspensions, epithelial SP fraction (n = 8) and stromal SP fractions (n = 8) separately) which were pooled in pairs at the RNA levels. Four microarrays were analyzed per group.

All the samples were hybridized in the Whole Human Genome Oligo Microarray (Agilent Technologies, Madrid, Spain) which encompassed more than 44000 human DNA probes. The protocols for sample preparation and the hybridization of the endometrial samples were adapted from the Agilent Technical Manual. Finally, hybridized microarrays were scanned in an Axon 4100A scanner (Molecular Devices, Sunnyvale, CA, USA), and the data were extracted with the GenePix Pro 6.0 software (Molecular Devices, Sunnyvale, CA, USA).

### Bioinformatic processing and data analysis

The GenePix Pro 6.0 software was used for array image analysis and for the calculation of spot intensity measurements, which were considered to be raw data. The gene expression profile was determined by comparing the experimental groups with the control group (2 by 2 comparisons) with non parametric tests. Two criteria were used to define the genes that had altered the mRNA abundance among the different sample sets: an absolute fold change of 2.0 or more, and a corresponding fold change p-value lower than 0.05.

### Functional analysis

To detect activations or inactivations in the biological functions or pathways, we used the Database for Annotation, Visualization and Integrated Discovery (DAVID) [34]. DAVID can search blocks of functionally-related genes by different criteria such as the Gene Ontology terms KEGG pathways, among others.

### Bioinformatic analysis of the gene expression

In order to remove all the possible sources of variation of a non biological origin between the arrays, densitometry values were normalized using the quantitative normalization function and were further transformed to the logarithmic scale (log2). Statistically significant differences between groups (SP epithelial versus total epithelial fraction, and SP stromal versus total stromal fraction) were carried out using non parametric rank products [35] method performed using the Bioconductor (http://www.bioconductor.org/) package RankProd, which was run using the R software (http://www.r-project.org/). Venn diagrams were drawn with Venny [36], and functional annotations were carried out using Ingenuity (http://www.ingenuity.com/), where the gene symbols and the fold changes of the up- and down-regulated probes were imported. Finally, probes as possible specific markers of the different cell populations were selected after running the J48 pruned tree classification algorithm implemented in the WEKA data mining software [37].

### Array Validation

To verify the results obtained by the cDNA microarray, real-time PCR was performed for three selected genes (n = 3, SP epithelium and stroma, total fraction of epithelium and stroma): *TNFAIP3* (Tumor necrosis factor, alpha-induced protein 3) as one up-regulated gene in the SP cells fraction, while *HIST1H1A* (Histone cluster 1, H1a) and *VWF* (Von Willebrand factor) were used as the down-regulated genes in the SP cells fraction. The gene expression levels from the endometrium were determined by real-time PCR using specific primers for the three genes: *TNFAIP3* Fw-5′-CACGCTCAAGGAAACAGACA-3′ and Rv-5′-TTCAAAGGGGCGAAATTGGA-3′; *HIST1H1A* Fw-5′- CTCGGGTTCCTTCAAGCTCA 3′ and Rv-5′-TCTTTGGCTTCGTCACCCTA-3′ and *VWF* Fw-5′-AACAGAGTGACAGTGTTCCCTA-3′ and Rv-5′ GACCCGATGACTCTTCAGCA-3′.

Real-time PCR was performed using the Roche LightCycler 2.0 (Roche, Mannheim, Germany) and the FastStart DNA Master SYBR-green 1 system according to the manufacturer's instructions. The glyceraldehyde 3′phosphate dehydrogenase (*GAPDH*) was used like control, the mean *GAPDH* concentration was used to normalize all the other genes tested from the same cDNA sample. The relative change in gene expression was recorded as the ratio of the normalized target concentrations in the cDNA dilution.

### Immunophenotypic characterization

The SP cells from the epithelial and stromal fractions were analyzed and characterized in comparison to the complete cellular fraction prior to Hoechst treatment.

The antibodies and concentrations used were: CD90 (10 µl, Chemicon International; Anti-Thy-1, clone F15-42-1, CBL415P); CD105 (10 µl Chemicon International; Anti-Endoglin, clone 8E11, CBL418F); CD34 (10 µl Chemicon International; Anti-CD34 Class II, Clone QBEND/10, CBL496P); CD45 (15 µl Chemicon International; Anti-CD45, clone F10-89-4, MAB4205F) and BCRP-1 (15 µl Chemicon International; Anti-BCRP, clone 5D3, MAB4155F).

### RNA isolation and reverse transcription

RNA extraction was performed according to the method described previously. Reverse transcription (RT) was carried out using the Advantage RT for PCR kit (Clontech, Palo Alto, CA, USA). The mastermix per sample was prepared as described in previous studies [23].

### PCR and Nested-PCR analysis for OCT-4, c-KIT, BCRP1, MDR1 and GAPDH genes

The sequence of the primers used were: OCT4 (also known POU5F1) fw-5′-AGAAAGCGAACCAGTATCGA-3′ and rev-5′-AGTACAGTGCAGTGAAGTGA-3′; c-KIT fw-out 5′-AAGCAGGAAGATCATGCAGA-3′ and rev-out 5′-AGGATATTTCTGGCTGCCAA-3′, fw-in 5′-TTCAAAGGAGTCTTCCTGCA-3′ and rev-in 5′-CTGGTAAGAAAAGCTCAGCA-3′; BCRP1 (also known ABCG2) fw-5′-CAAGCAGGATAAGCCACTCA-3′ and rev-5′-CAGCTCTGTTCTGGATTCCA-3′; MDR1 (also known ABCB1) fw-out 5′-TTGCAAATGCAAGAGGAGCA-3′ and rev-out 5′-ATTTTCACGGCCATAGCGAA-3′, fw-in 5′-TTGAAGGGTCTGAACCTGA-3′and rev-in 5′-CAATACAGGTTCCTGACTCA-3′. GAPDH expression was used as RNA as a housekeeping gene for normalization and the primer sequence used was to forward 5′-GAAGGTGAAGGTCGGAGTC-3′ and reverse 5′-GAAGATGGTGATGGGATTTC-3′. GAPDH, OCT-4 and BCRP1 expression was detected in the first round of PCR while c-KIT and MDR1 were detected in the second round of the nested-PCR.

The molecular analyses of these genes were carried out in human SP fraction versus NSP fraction of epithelial and stromal compartments. The human ESC line VAL-4 was used as a positive control and distilled water was used as a negative control.

PCR and nested-PCR were carried out as described in our previous molecular analysis [23] using a BIOMETRA thermal cycler. All bands were isolated and sequenced to verify the identity of the PCR products.

### Telomerase activity detection assay

Telomerase activity was analyzed using the TRAP_EZE_ ® Telomerase Detection Kit (Chemicon), and further staining was done with SYBR® Green I (Molecular probes). Briefly, fresh cells (1000–5000 cells) were harvested, washed once in Ca^2+^/Mg^2+^-free PBS, and were immediately resuspended in lysis buffer. After treatment on ice and spinning at high speed, samples were subjected to a PCR reaction following the manufacturer's instructions. PCR products were run in polyacrylamide gel (BioRad) under non denaturing conditions, and amplified fragments were then stained in SYBR green for visualization in a transilluminator. Each experiment included the human embryonic stem cell line, VAL-4, as a positive control [38], and the NSP population as a negative control.

### Clonogenicity assays under hypoxic conditions

Endometrial SP and NSP cells were seeded in triplicate at different clonal densities (10-20-200 and >600 cels/cm^2^) and were cultured in DMEM/F-12 (Sigma-Aldrich, Spain) containing 10% FBS, 2 mM glutamine (Invitrogen) and antibiotic-antimycotic under hypoxia (2% O_2_, 37°C, 5% CO_2_, 90% humidity) [29]. After 15 days cultures were fixed and stained with toluidine blue. The cloning efficiency (CE) was determined from the formula CE (%) = (number of colonies/number of cells seeded) * 100. The mean and standard errors of the mean (SEM) were obtained from each clonal density described above, and the overall mean (all the media of the different clonal densities) and the overall SEM were calculated for each cell type (epithelial SP, stromal SP, epithelial NSP, stromal NSP). Statistical analysis of the data was performed by a Student's t-test. A p-value of 0.05 was considered statistically significant.

Moreover hypoxic clonal assays were performed before the Hoechst treatment (epithelial and stromal fresh cells suspensions) in order to compare with the cloning efficiency described in SP and NSP cells under hypoxic conditions.

### *In vitro* differentiation

The SP/NSP cells from the epithelial and stromal fractions were cultured *in vitro* with adipogenic and osteogenic differentiation media. Briefly, cells were maintained under normoxic conditions (18–20% O_2_, 37°C, 5% CO_2_, 90% humidity) during the protocol of differentiation. SP and NSP cells were treated with the adipogenic induction media (Lonza, Barcelona, Spain) (h-insulin, L-Glutamine, MCGS, Dexamethasone, Indomethacin, IBMX and Penicillin/streptomycin) and the osteogenic induction media (Lonza) (Dexamethasone, L-Glutamine, Ascorbate, MCGS, Penicillin/streptomycin and β-Glycerophosphate) for two-three weeks according established protocols by Lonza. Moreover, the SP and NSP cells were treated with only the maintenance media (the technique's negative control). Afterward, cells were fixed and the adipogenic/osteogenic differentiation was detected with Oil Red O staining and bone sialoprotein immunocytochemistry. The adipogenic differentiation was demonstrated at mRNA levels with specific primers for lipoprotein lipase (LPL) [39]. Also, the osteogenic differentiation was assessed at mRNA levels with the expression of Osterix, an osteoblast marker [40]–[41]. Bone sialoprotein (BSP, MAB1061, Chemicon Internetional) immunocytochemistry was performed in order to verify osteogenic differentiation in SP vs NSP cells [40]–[41].

RNA extraction and real-time PCR were performed as previously described. Our positive controls for Oil Red O and LPL expression were adipocyte cells cultured from tissue explants. Positive controls for bone sialoprotein expression and genes battery were osteocytes removed from bone explant. In both cases the negative controls were non-sorted endometrial cells.

### Ethics Statement

Procedures performed on animals were approved by Unidad Mixta Universidad de Valencia UVEG-CIPF and Instituto Valenciano de Infertilidad (IVI) review board. Mice were maintained in specified pathogen free (SPF) facilities and conditions and animal breeding was carried out “ad libitum”.

### Animal Model: Xenotransplantation assays

NOD-SCID mice ((strain code 394; NOD.CB17-Prkdc^scid^/NCrCrl) (Charles River Laboratories, Spain)) were used for xenotransplantation experiments. The SCID mutation was transferred onto a non obese diabetic background; animals had impaired T and B cell lymphocyte development. Female mice at 5–6 weeks were injected with our candidate cells and a subcutaneous estrogen pellet was implanted.

The hypoxic epithelial and stromal SP/NSP single-cell suspensions (100,000–500,000 cells) in 5–10 µl of DMEM-matrigel (Sigma-aldrich, Spain) (1∶1) were injected into the immunosuppressed mice subcutaneously on the left flank. At transplantation, the NOD-SCID mice were also implanted subcutaneously with E_2_ pellets at the neck level (SE121, 17β-estradiol 0.18 mg/60 days; Innovative Research of America, Sarasota, FL). These xenotransplanted mice were sacrificed according to the experimental protocol 60 days after the injection.

### Laser Capture Microdissection

Subcutaneous tissues from the mice injected with SP cells were excised, frozen in isopentane over liquid N_2_, and stored at −80°C. Frozen tissues were processed in cryotome at −20°C and sectioned at 5–7 µm; sections were finally adhered to sterile RNA free, uncharged and uncoated glass slides (Leica Slides, Wetzlar, Germany). Then, they were stained with HistoGene LCM (Arcturus Bioscience Inc., CA, USA).

LCM (IM 1000 icon, Leica Microsystems) was used to isolate the epithelial glands from the mice-derived subcutaneous tissue samples.

### Molecular analysis

The dissected glands were ejected off the slide with a single defocused laser pulse, and were catapulted directly into the cap of a microfuge tube containing extraction buffer (Picopure RNA Isolation Kit, Arcturus Bioscience Inc., CA, USA). Subsequently, the dissected glands in the extraction buffer solution were heated at 42° for 30 minutes to obtain a cell lysate. The RNA isolation was carried out at room temperature with the PicoPure RNA Isolation Kit (Arcturus Bioscience Inc., CA, USA).

Quantitative real-time PCR analysis was performed as described previously. The oligonucleotide primer sequences for human *GAPDH* (Hu*GAPDH*) were Fw-5′-ACACTCAGACCCCCACCACA-3′ and Rv-5′- CATAGGCCCCTCCCCTCTT-3′ and mouse *Gapdh* (Ms*Gapdh*) were Fw-5′-AACTCGGCCCCCAACACT -3′ and Rv-5′-CCTAGGCCCCTCCTGTTATTATG-3′ [42].

Negative control without a cDNA template was run with every assay to assess overall specificity. Furthermore, a negative control was performed with the non specific primers, human DNA was amplified with the mouse primers, and mouse DNA was amplified with human primers. The positive control was achieved with the cDNA from the human endometrium for human primers, and with the cDNA from mouse uteri for the mouse primers. The identity of the PCR products was verified by DNA sequencing.

### Immunohistochemistry of human endometrial-like tissue from SP in NOD-SCID mice

Tissue sections from human endometrium (positive control), mice tissue (negative control) and putative human endometrial glands surrounded by mice adipose tissue in the injection site were analysed using immunohistochemistry in order to localize Human Progesterone Receptor (Hu-PR-FITC, Abcam ab 27616-500, lot 688384).

After deparaffination and rehydration, sections were rinsed in citrate buffer (pH 6, 95°C) for antigen retrieval. Then, slides were incubated overnight at 4°C with anti-Hu-PR-FITC (Final concentration: 1/25) and counterstained with 6-diamidino-2-phenylindole (DAPI).

Immunolocalization of the specific cell populations corresponding to human endometrial cells in the mice injection site was visualized and photographed using an Olympus 35 mm camera attached to a fluorescence microscope (Olympus Provis AX70, Olympus, Optical España S.A.).

### Statistical analysis

The statistical analysis of the stromal and epithelial SP cells in the human endometrium of women of different ages was performed by a Student's t-test. A p-value of 0.05 was considered statistically significant.

## Results

### SP isolation in epithelial and stromal fractions of the human endometrium

**Figure 1:**
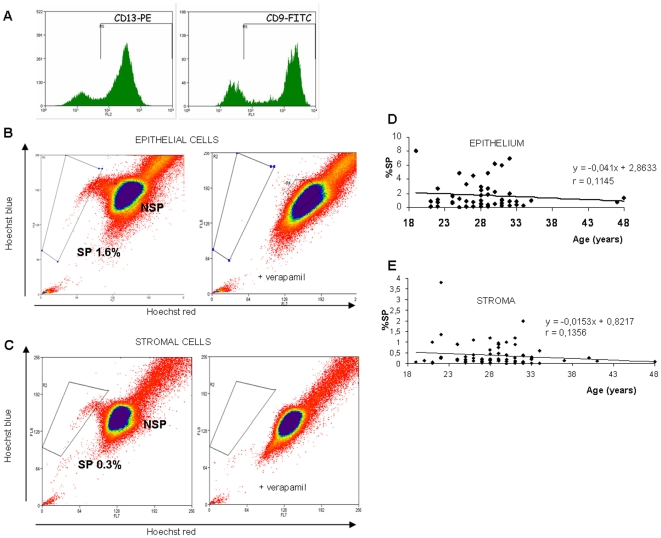
Isolation of purified epithelial and stromal SP cells. (A) Purity of the stromal and epithelial cell fractions was assessed by immunocytochemistry and flow-cytometry, 79.6% of the cells were CD13+ (stroma), and 75.8% were CD9+ (epithelium). (B & C) Distribution of the SP and NSP populations of living cells isolated from the epithelium (B), and stroma (C) of the human endometrium. (Right) coadittion of 100 µm verapamil resulted in the disappearance of the SP fraction. (D & E) Correlation tables showing the percentage of SP in the epithelium (D) and stroma (E) through womeńs reproductive life.

We first tested both fractions of the human endometrium for the presence of SP cells. As shown in Figure 1A, the purity of the stromal and epithelial cells was assessed by immunocytochemistry and flow cytometry, which confirmed that 79.68% of cells were CD13+ in the stromal compartment, and that 75.89% were CD9+ in the epithelial fraction. Moreover, the cell viability after the separation of both fractions ranged from 60% to 85%, and enabled the Hoechst protocol to be carried out without further *in vitro* culture. In general, the cell viability diminished by around 10% after Hoechst incubation.

With the indicated features of purity and viability, we isolated the SP from the epithelial (Fig. 1B) and stromal (Fig. 1C) compartments of the human endometrial samples and found that these cells represent 1.68±0.27% of the total living cell population in the epithelium (n = 50) and 0.39±0.06% of the stromal fraction (n = 78). In all cases, the identification of SP cells was determined by the addition of 100 µm verapamil, an ABCB1 inhibitor (Fig. 1 B&C). Furthermore, no significant difference was found in either the percentage of epithelial (n = 50) or the stromal (n = 78) SP throughout reproductive life (Fig. 1 D&E), thus supporting the concept of quiescent and slow cycling cells [23].

### Endometrial SP Gene Signature

In order to acquire knowledge about the genetic footprint of this cell subpopulation, we generated the endometrial SP gene signature. A comparative wide genomic analysis of the epithelial SP (n = 8) and the stromal SP (n = 8) versus their corresponding total endometrial epithelial (n = 8) and stromal fractions (n = 8) was performed. RNA samples were pooled in pairs by analyzing a total of 4 pool samples per arm.

**Figure 2:**
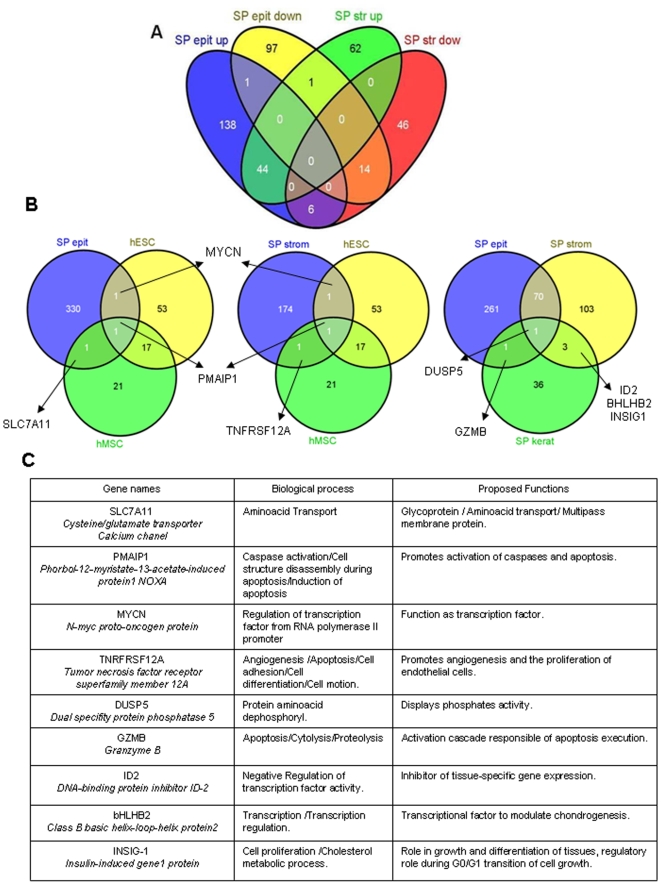
Endometrial SP Gene Signature. (A) The Venn diagram represents the numbers of shared genes up- and down-regulated in the epithelial and stromal SP of the human endometrium (see Table S1 for details). (B) Venn diagrams representing: (left) the number of shared genes expressed by epithelial SP, human embryonic stem cells (hESC), and human mesenchymal stem cells (hMSC). (Center) shared genes expressed by the stromal SP, hESC and hMSC. (Right) the Venn diagram represents the number of shared genes expressed by epithelial SP, stromal SP and SP from the keratinocytes (see Table S3 for details). (C) Table describing the biological process and proposed functions of the consensus genes obtained in the previous Venn diagrams.

Using the pre-defined criteria of the p-value lower than 0.05, we identified a total of 196 up- and 117 down-regulated genes which were differentially expressed in the epithelial SP versus the whole epithelium, and also in 121 up- and 73 down-regulated genes in the stromal SP versus the complete stromal compartment (Fig. 2A, Table S1). The stromal and epithelial SP top ten up- and down- regulated genes, along with their functionality, are presented in Table S2. Interestingly 44 genes were commonly up-regulated and 14 were down-regulated in both the epithelial and stromal SP, indicating a common cell fate irrespectively of the compartment providing an endometrial SP stem cell gene signature (Fig. 2A, Table S3).

The raw data files of current experiments are uploaded to the NCBI Gene Expression Omnibus (GEO) database with number GSE21633.

In order to understand how cellular functionalities are differentially related in the SP versus their original endometrial fraction, we analyzed their functional profiles with Ingenuity Pathway Analysis software (http://www.ingenuity.com). The stromal SP was enriched in genes involved in cell death, cell cycle, cell-to-cell signaling and interaction, cellular growth, proliferation, and cellular movement compared to its whole fraction; in the epithelial SP however, the genes involved in cell-to-cell signaling and interaction, cellular growth and proliferation, cellular movement and cell death were identified.

In the epithelial SP, the top canonical pathways identified were TREM1 signaling, glucocorticoid receptor signaling, dendritic cell maturation, the role of cytokines in mediating communication between immune cells and natural killer cell signaling; the top networks implicated were cancer, tumor morphology, connective tissue disorders and hematological system development and function, among others. In the stromal SP, the top canonical pathways were TREM1 signaling, glucocorticoid receptor signaling, the role of cytokines in mediating communication between immune cells, VDR/RXR activation and the role of pattern recognition receptors in recognition of bacteria and viruses; the main networks implicated were hematological system development and function, cell cycle, cell death, hematological disease, tissue development and cellular and growth proliferation, among others.

The comparative genomic analysis from the epithelial and stromal SP versus the published information about human mesenchymal stem cells (hMSC) and human embryonic stem cells (hESC) [43] revealed that phorbol-12-myristate-13-acetate-induced protein 1 (PMAIP1/APR/NOXA) was the only gene regulated in all three mesenchymal stem cell populations, and was also found to be common for both endometrial SP, together with the tumor necrosis factor receptor superfamily member (TNRFRSF12A) and the cysteine/glutamate transporter calcium channel resistance protein SLC7A11 (Fig. 2B&C). Interestingly, the N-myc protooncogen protein was the only gene shared between the human endometrial SP and hESC, but it was not present in hMSC (Fig. 2B&C).

The comparison of the gene list between the endometrial SP (stromal and epithelial) and the published information obtained in SP keratinocytes [28] revealed that there was one gene in common for the three different SP populations, this being the so-called Dual specific phosphatase 5 (*DUSP5*) (Fig. 2B&C).

Real-time PCR of genes described in materials and methods section validated the results obtained by microarrays.

### Phenotype of epithelial and stromal SP and Telomerase Activity

**Figure 3:**
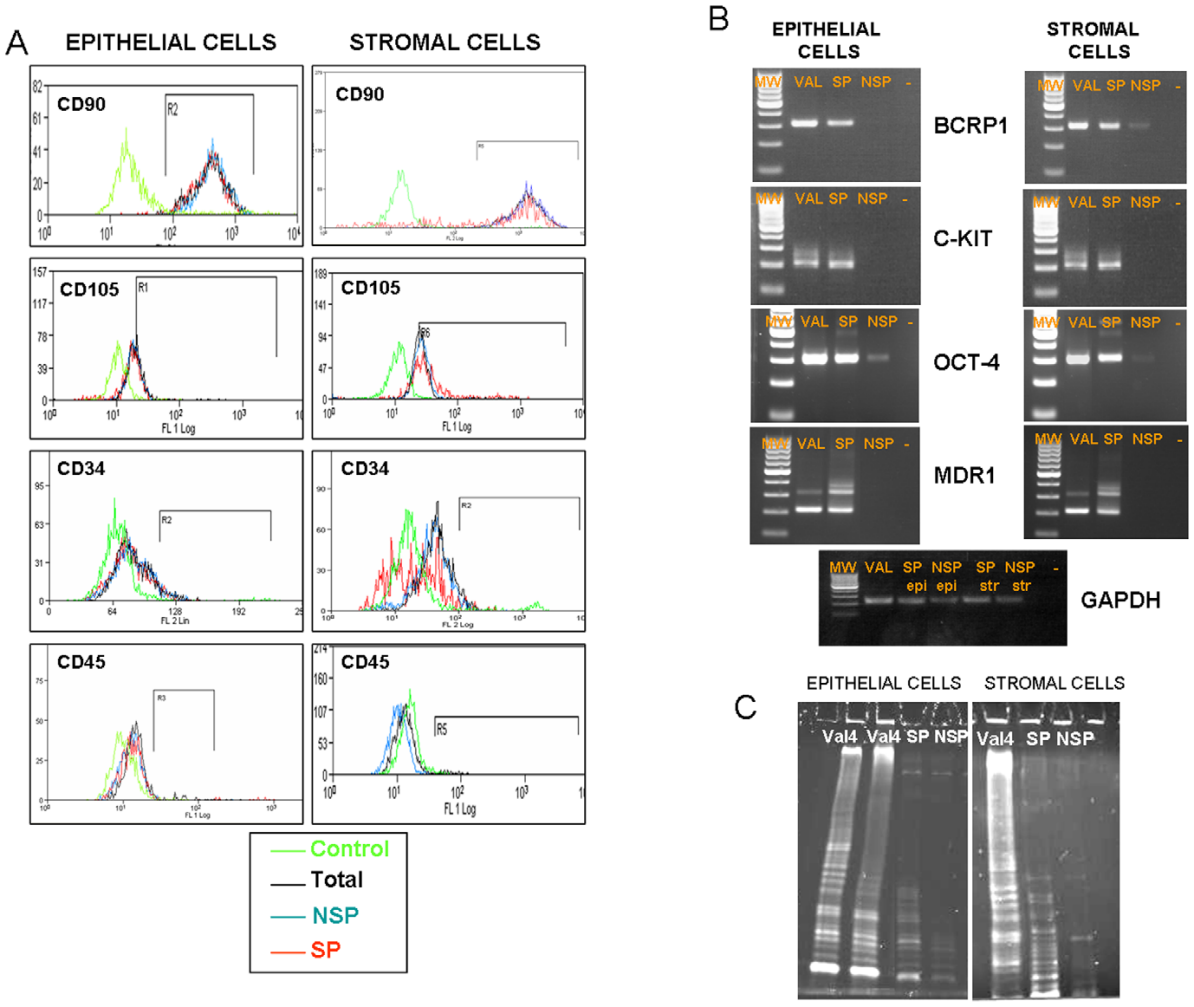
Phenotype of epithelial and stromal SP and telomerase activity. (A) Sorted SP, NSP and total endometrial fraction for epithelium and stroma were fixed and positive cells were quantified by FACS for mesenchymal markers (CD90 & CD105) and hematopoietic markers (CD34 & CD45). (B) Sorted SP versus NSP cells were analyzed by PCR and nested-PCR showing the bands corresponding to typical undifferentiated markers c-KIT and OCT-4 as well as Side Population/ABC transporter markers like BCRP1 and MDR1. VAL corresponds to the human embryonic stem cell line VAL-4 obtained from the Spanish Stem Cell Bank (http://www.isciii.es/htdocs/terapia/terapia_lineas.jsp). (C)Telomerase activity of sorted SP and NSP from the stromal and epithelial compartments are presented in this figure having as a positive control VAL-4. In both compartments SP depicted an intermediate telomerase pattern compared to hESC and NSP.

We next characterized the phenotype of SP in comparison with the NSP cells and the whole endometrial compartment that they originate from. The immunophenotype was based on the flow cytometric analysis of a subset of mesenchymal stem cell markers (CD90 and CD105), hematopoietic markers (CD34 and CD45) and ABC transporters (BCRP1) (Fig. 3A).

The flow cytometric analysis (n = 6) showed that 70% of the epithelial SP cells and 80% of the stromal SP cells were positive for CD90. Only 1–2% of cells were positive for CD34, 5–7% were positive for CD45, and 4% were positive for CD105. Taken together, these results strongly suggest that the stromal and epithelial SP have a mesenchymal phenotype [39].

Comparison at mRNA levels between epithelial/stromal SP versus NSP cells demonstrated an enriched expression pattern of typical undifferentiated markers (c-KIT and OCT-4), and specific ABC transporters markers (BCRP-1 and MDR1) in SP cells (Fig. 3B). Interestingly, only 15–17% of the SP cells from the epithelial and stromal fractions were positive for the typical SP marker BCRP1 at the protein level.

Moreover, endometrial epithelial and stromal SP present an intermediate pattern of telomerase activity compared to undifferentiated hESC with a ladder of amplification products of 6 base increments starting at 50 nucleotides and NSP, or the total endometrial compartment, suggesting that SP cells are an intermediate population in terms of the telomerase length between pluripotent and oligopotent cells (Fig. 3C).

### Cell Culture under Hypoxic Conditions

After several attempts, we realized that endometrial SP cells did not proliferate adequately under normoxic conditions. So we decided to follow-up the hypoxic approach described to culture human myometrial SP cells under 2% oxygen tension [29]. Initially, we tested the cloning efficiency of the isolated epithelial fraction being 1.43%±0.72% (n = 3) and the stromal fraction was 3.94%±1.25% (n = 3), corresponding to the 6.5 and 3.1 fold increases, respectively, than those reported under normoxic conditions for the indicated fractions [14].

**Figure 4:**
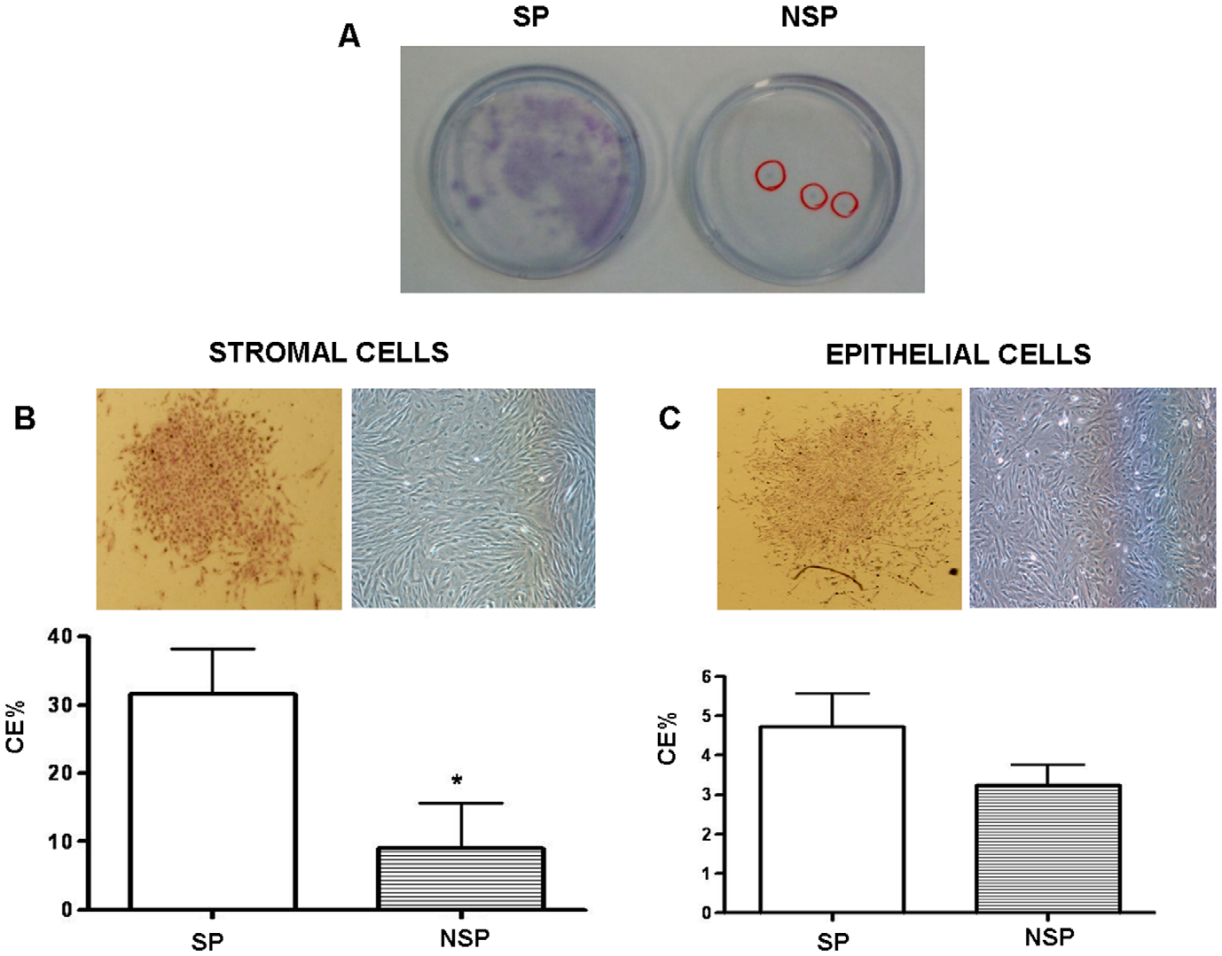
Cloning efficiency under hypoxic conditions. (A) Human endometrial cell clones under hypoxic conditions. Typical colonies formed by sorted SP (left) and NSP (right) human endometrial stromal cells were seeded at clonal densities and cultured for 15 days under hypoxic conditions. (B) Colony morphology (upper panel) and cloning efficiency (%) of sorted stromal SP that was significantly greater than NSP cultured in hypoxic conditions (p = 0,018). (C) Colony morphology (upper panel) and cloning efficiency (%) of sorted epithelial SP versus NSP cultured in hypoxic conditions. (Data are means ± SEM)

Under hypoxic conditions, clonogenicity was significantly higher in stromal SP (31.6±6.6% (n = 37)) versus NSP (9.06±6.6% (n = 36)) (p value = 0,018). However, the differences were not statistically significant in the epithelial compartment corresponding to 4.71±0.8% (n = 29) for SP versus 3.24±0.5% for NSP (n = 28) (p value = 0,157) (Fig. 4).

### Adipogenic and Osteogenic Differentiation of Epithelial and Stromal SP

**Figure 5:**
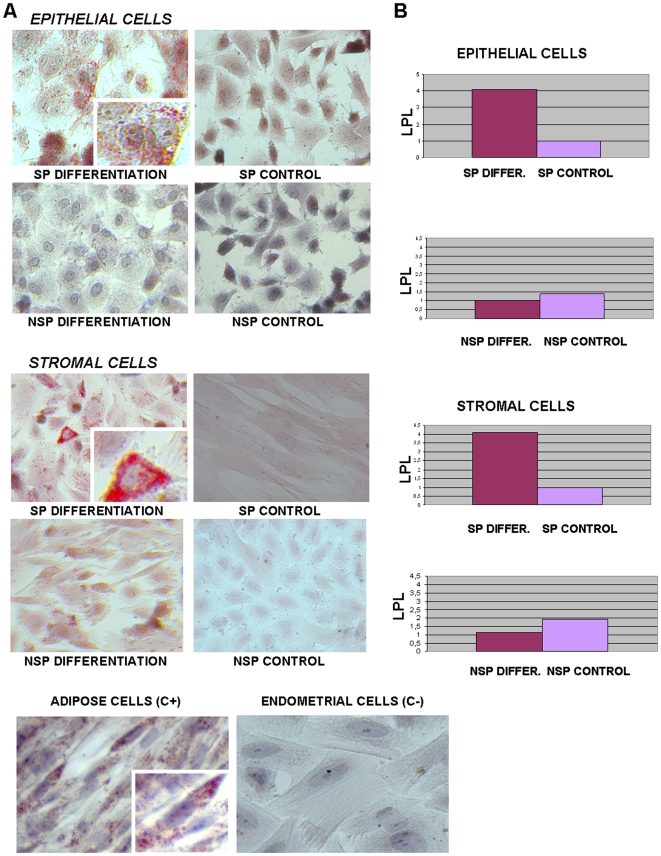
Adipogenic differentiation *in vitro* of epithelial and stromal SP. (A) Induction of adypocite differentiation of epithelial and stromal SP, but not NSP, determined by Oil Red O staining of lipids droplets. Staining with Oil Red O revealed the presence of lipids vacuoles in the sorted endometrial SP cells versus NSP and their controls cultured without differentiation media.Positive control are adipose cells and negative control are non-sorted endometrial cells (lower panel). (B) Induction of adypocite differentiation of epithelial and stromal SP, but not NSP, determined by real-time PCR of adipocyte-specific gene lipoprotein lipase (*LPL*) demonstrated differential gene expression of SP versus NSP and their controls.

We examined the potential of SP cells for multilineage differentiation *in vitro*. We cultured and expanded the SP and NSP cells of both the epithelial and stromal compartments under hypoxic conditions for 2–4 weeks. Then cells were cultured in an adipogenic induction media (see Materials and Methods) versus a control medium, and were further cultured under normoxic conditions for 2 more weeks. In the presence of adipogenic induction media, SP, but not NSP, showed adipogenic differentiation as assessed by the presence of lipid vacuoles stained with Oil Red O (Fig. 5A) and by the increase of the lineage-specific gene LPL at the mRNA levels (Fig. 5B) in the SP cells treated with the induction media versus those treated with the control media [39].

**Figure 6:**
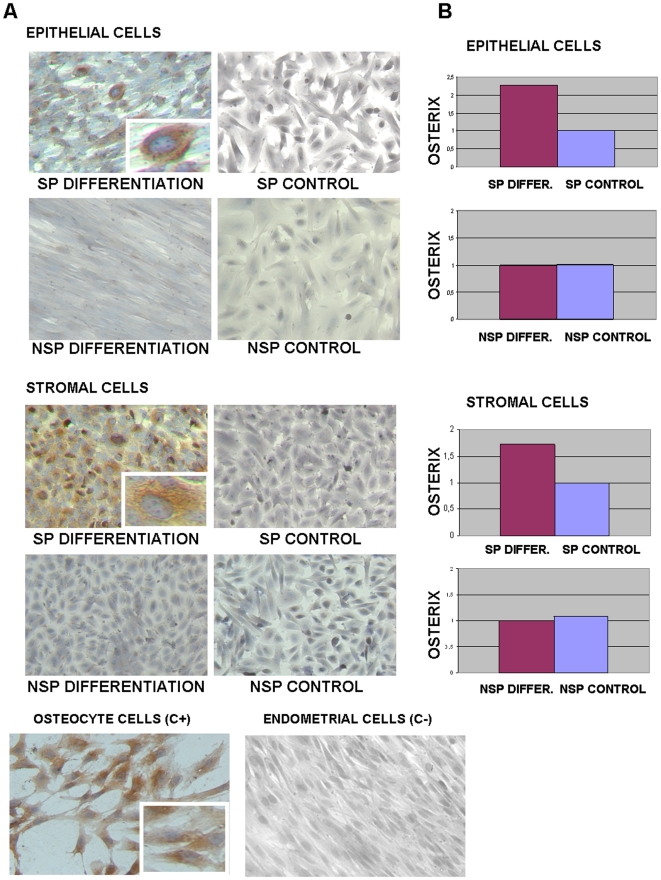
Osteogenic differentiation *in vitro* of epithelial and stromal SP. (A) Induction of osteogenic differentiation of epithelial and stromal SP, but not NSP, determined by immunocytochemistry with bone sialoprotein. Staining with a protein highly specific for bone revealed the presence of osteoblast in the sorted endometrial SP cells versus NSP and their controls cultured without differentiation media. Positive controls are osteocyte cells from explants and negative control are non-sorted endometrial cells (lower panel). (B) Induction of osteocyte differentiation of epithelial and stromal SP, but not NSP, determined by real-time PCR of osterix and osteonectin demonstrated differential gene expression of SP versus NSP and their controls.

After hypoxic conditions, SP vs NSP cells were also cultured in an osteogenic induction media (see Materials and Methods) versus a control medium. Osteoblast differentiation was demonstrated by the positive staining for bone sialoprotein (BSP) (Fig. 6 A) and confirmed at RT-PCR level by the increased expression of osteoblast marker osterix. The expression of cbfa-1 was also analyzed in the same cells, but not significant differences were observed, showing preosteoblast features instead of the fully differentiated osteoblast. (Fig. 6B) [40]–[41].

### Reconstruction of human endometrial-like tissue from SP in NOD-SCID mice

**Figure 7:**
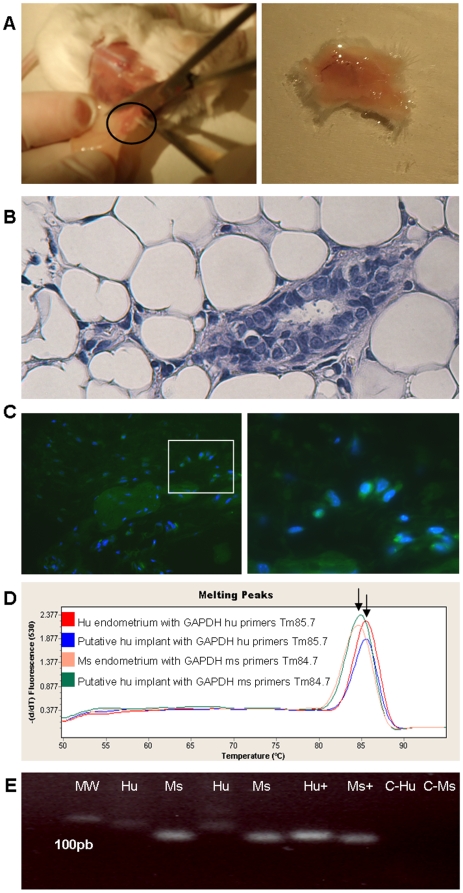
Reconstruction of human endometrial-like tissue from SP in NOD-SCID mice. (A) Macroscopic finding of the injected site of sorted SP cells sixty days after transplantation in NOD-SCID mice showing putative endometrial like implant in subcutaneous back tissue. (B) H&E staining of putative human endometrial glands surrounded by mice adipose tissue in the injection site. (C) Left, immunohistochemical analysis for Human Progesterone Receptor (Hu-PR) in endometrial like structures in mice subcutaneous tissue obtained after injection of SP cells from human stroma (40X). Right, Detail of green fluorescent signal due to Hu-PR co-localized with DAPI in a putative human gland (High magnification: 63X-oil immersion microscopy). Negative controls with deletion of the antibody and using mice tissue were satisfactory. (D) Real Time diagram showing melting peaks. The difference between both Melting Temperatures (Tm) was an indicator of the two existing amplicons (specific nucleotide sequence) corresponding to the two different species (human and mouse). (E) m-RNA expression of human *GAPDH* (79 bp) and mouse *GAPDH* (75 bp) in reconstructed human tissue determined by real-time PCR. MW (lane 1): molecular weight pattern. Hu (lane 2 and 4): laser microdissected glandular tissue extracted from xenotransplanted mice hybridized with human primers. Ms (lanes 3 and 5): laser microdissected glandular tissue extracted from xenotransplanted mice hybridized with mice primers. Hu+ (lane 6): human endometrial tissue hybridized with human primers. Ms+ (lane 7): mouse endometrium hybridized with mice primers. C-Hu (lane 8): human endometrial tissue hybridized with mice primers. C-Ms (lane 9): mouse endometrium hybridized with human primers. All bands were sequenced confirming the human tissue presence in the laser microdissected glands extracted from mouse adipose tissue.

SP and NSP cells (100,000–500,000) were cultured in 2% O_2_ for 15 days, suspended in 5–10 µl of DMEM-MATRIGEL (1∶1) and then injected in the NOD-SCID mice subcutaneously on the left flank. At transplantation, mice were also implanted subcutaneously with E_2_ pellets at the neck level (SE121, 17β-estradiol 0.18 mg/60 days). In total, 50 animals were injected subcutaneously with epithelial NSP, stromal NSP, epithelial SP and stromal SP cells having as negative control the injection of Matrigel and as a positive control tumorigenic leukemic cell line V937. The positive and negative controls confirmed the presence or absence of growing cells at the injection site. In all animals, NSP injection from epithelial and stromal origin and SP injection from epithelium didn't give rise to endometrial-like structures. However, in 1 animal in which SP cells from the stromal fraction were injected, a vascularized macroscopic structure in the subcutaneous fat at the injection site was observed (Fig. 7A). Furthermore, at the histological level, structures compatible with the endometrial glands and stroma were demonstrated (Fig. 7B) and the human origin of these structures was proved by molecular and immunohistochemichal analysis (Fig. 7C–D–E).

Therefore, putative human endometrial glands surrounded by mice adipose tissue in the injection site expressed Human Progesterone Receptor (Hu-PR-FITC) confirming the endometrial-like tissue from SP in the mice model (Fig. 7C).

To confirm the human origin of these structures, the subcutaneous tissue of the NOD-SCID mice was frozen and sectioned, and endometrial-like structures were dissected-out using laser capture. Extracted mRNA was analyzed by real-time PCR analysis with oligonucleotide primer sequences for human glyceraldehyde 3′phosphate dehydrogenase (Hu*GAPDH*) and mouse *Gapdh* (Ms*Gapdh*), which had been designed specifically to distinguish human cells from mice cells (see Materials and Methods) [42]. The difference between both melting temperatures (Tm) was an indicator of the two existing amplicons (specific nucleotide sequence) corresponding to the two different species (human and mouse) (Fig. 7D) and moreover bands were also visible in agarose gel electrophoresis (Fig. 7E), and the identity of the PCR products was verified by DNA sequencing.

## Discussion

The results of this study strongly suggest that the endometrial SP cells from both the stromal and epithelial compartment are putative human endometrial stem cells. These cells have a mesenchymal origin, and have the ability to differentiate to adipocytes and osteocyte *in vitro* and to give rise to endometrial-like tissue *in vivo*.

We demonstrate that the proportion of SP cells in whole epithelial and stromal fractions ranged from 0.06–6.2% and 0.01–3.8%, respectively, which is in accordance with published works [21], [32]. We also conclude that this population remains stable throughout reproductive life, thus supporting the concept that these cells are slow cycling, as suggested in the murine model [22], [23]. This stable population could also be responsible for the re-growth of the human endometrium in postmenopausal women after adequate hormone treatment.

Gene expression patterns provide a common language among biological phenomena and cell subpopulations, and allow an alternative approach to infer molecular mechanisms for complex functions. Our experimental design allowed us to identify 44 genes commonly up-regulated and 14 down-regulated in both epithelial and stromal SP, indicating a common cell fate irrespectively of the compartment providing an endometrial SP stem cell gene signature (Table S3). This is an important finding to develop a set of markers for the identification of human endometrial SP.

The phorbol-12-myristate-13-acetate-induced protein 1 (PMAIP1/APR/NOXA) was the only gene regulated in hMSC and in both endometrial SP. This gene is a pro-apoptotic member of the *BCL-2* family which is up-regulated by the p53 promoting activation of caspases and apoptosis. The *N-MYC* protooncogen was the only gene shared among the human endometrial SP and hESC, but was not present in hMSC (Fig. 2B–C). Finally, Dual-specific phosphatase 5 (*DUSP5*) was the only gene to be regulated among endometrial SP (stromal and epithelial) and the SP from keratinocytes. It represses members of the mitogen-activated protein kinase which are associated with cellular proliferation and differentiation. In the final list of 507 genes provided in supporting information Table S1, we have identified 45 genes in epithelial SP and 30 genes in stromal SP implicated in stem cell functions. Only 19 genes were common in both epithelial and stromal SP: GDF15, IL6, IL1B, IL8, CXCL1, CXCL2, ICAM1, GADD45A, IL23A, HSPA1A, BCL2A1, HES1, CCL3, CD69, MYCN, XCL1, MMP3, INHBA y CSF2. Specifically, GDF15 (Growth differentiation factor 15) is a member of the transforming growth factor-beta superfamily and regulate tissue differentiation and maintenance. Also GDF15 is associated with tissue remodelling events in reproductive processes [44] suggesting the putative role in endometrial repair following menstruation. IL8 (interleukin 8) is a chemokine mediator of the inflammatory response nevertheless this molecule is implicated in some biological process related to the quiescence status of somatic stem cells like cell cycle arrest and negative regulation of cell proliferation. Participation of GADD45A (growth arrest and DNA-damage-inducible alpha) in mammalian epithelial tissues involve the dynamic regulation of DNA methylation patterns, required for progenitor maintenance and self-renewal in mammalian somatic tissue [45]. BCL2A (BCL2-related protein A1) up-regulated in epithelial and stromal fractions is associated with anti-apoptosis, it was described with a significant increased expression in the SP cells in human oral squamous cell carcinoma and related to cellular survival [46]. HES1 is regulated by NOTCH1, a membrane receptor implicated in maintaining 'stemness'. To note, notch-1, and its modulator MSI1 are expressed in the human endometrium [47], and have been linked to a putative dysregulation of endometrial stem cell function in endometriosis and endometrial carcinoma. MYCN (N-myc proto-oncogen protein) is a shared gene expressed also human Embryonic Stem Cells (hESC). MYCN is regulated by PTEN, gene involved in cellular growth and tissue differentiation. Specifically alteration of PTEN upregulates MYCN expression, which may be an important gene in endometrial carcinogenesis in the adaptation of hypoxia for survival conducted by the upregulation of HIF1 (hypoxia-inducible transcription factor 1) [48]. MYCN roles in cellular proliferation and hypoxic environment survival suggest endometrial stem cells functions of this gene.

Different groups [49]–[51] have reported the mesenchymal origin of the putative endometrial SSC based on the screening of specific mesenchymal and haematopoietic lineage markers. In general terms, our work is in agreement with those of Kato et al. [32] and Tsuji et al. [21] in that the immunophenotype of the endometrial SP population from both the epithelium and stroma strongly expressed CD90 and was negative for hematopoietic markers CD34 and CD45 [39], thus corroborating its mesenchymal origin. Nevertheless, some differences in other markers found could be due to a different pre-processing of the endometrial cells as in the case of Tsuji [21] where SP cells had to be cultured for 3 to 4 days in primary culture prior to the assay. In our case, the flow cytometry analysis was performed in freshly isolated SP and NSP cells (Figure 3A). Nevertheless, these results together with the genomic data strongly suggest that even though the endometrial SP population was of a mesenchymal lineage, it also expressed the *N-MYC* gene unlike classical hMSC and, therefore, SP could be a different subpopulation of hMSC cells.

Molecular comparison with typical stemness and ABC transporters markers between the epithelial/stromal SP vs. the NSP cells further verified their differential expression pattern in the SP fraction, thus confirming a specific SP signature. Although *BCRP1* and *MDR1* were expressed in the SP at the mRNA level, the genome wide analysis reveals that *ABCG2 (BCRP1)* and *ABCB1 (MDR1)* are not differentially overexpressed in SP versus the total fraction. A similar finding was described in the SP keratinocytes isolated from the skin [28], [52]. One explanation for this would be that arrays studies could show several genes with variable expression associated with ABC/MDR transporters in the total epithelial and stromal fractions versus their SP fractions. Nevertheless, the fold change difference may be not differentially relevant in the arrays (p-value<0.05) but detectable in RT-PCR. In general terms, however, the role of these transporters remains unclear, even in blood cells.

In differentiated cells, telomeres become shorter with each cycle of cell division, and telomerase activity decreases unlike undifferentiated cells such as hESC. The expression of the telomerase activity in the SP cells displays an intermediate pattern that suggests a putative SSC origin [53].

In our laboratory, the isolated endometrial SP cells were grown in normoxia for a few days losing their mesenchymal stem cell markers. This is in contrast with the results by Kato and collaborators who report a cell culture of SP of up to 5 months, and does not coincide with the work of Tsuji and collaborators who show greater colony-forming efficiency compared to NSP under normoxic conditions. Nevertheless, our work is in agreement with Ono and collaborators [29] who were unable to culture myometrial SP cells under normoxic conditions, which suggests that SSC may reside in a hypoxic niche. Under these conditions, our SP cells exhibit high proliferative capacity, and the cloning efficiency of the stromal SP was statistically significant compared than NSP, 31.6±6.6% (n = 37) *versus* 9.06±6.6% (n = 36), respectively.

Hypoxic environment in human endometrium was demonstrated to be present throughout the menstrual cycle [54]. Hypoxia is known to exert growth-promoting effects toward any cell type; nevertheless cloning efficiency is merely a preliminary tool to characterize a putative stem cell population that must be correlated with genomic and phenotypic putative markers and functional proof.

We have also shown that SP cells retain the capability to differentiate *in vitro* into different mesodermal lineages, including adipocytes and osteocytes [39]–[41]. The main challenge was to demonstrate that SP cells generated endometrial human tissue when injected into the subcutaneous tissue of immunodeficient mice (NOD-SCID). To this end, we have assessed in the single reconstructed endometrial tissue from stromal SP not only the human origin by the expression and sequencing of a human specific gene in the endometrial-like gland tissue obtained by laser capture microscopy, but also by the presence of the human progesterone receptor in this endometrial glands, which suggests that they are a bona fide functional human endometrium. Further supporting this concept, a recent study published by Kato et al in 2010 [55] demonstrated the different capability of SP versus NSP cells from human endometrial cancer to reconstitute solid, large and invasive tumors in an immunocompromised mice model.

In conclusion, we isolated and characterized the SP corresponding to the stromal and epithelial compartments on the basis of the endometrial SP gene signature, immunophenotyping and telomerase activity. We analyzed their clonogenic activity under hypoxic conditions and the *in vitro* differentiation capability to adipogenic and osteogenic lineages. Finally, reconstructed human endometrium was created after subcutaneous injection of SP cells in NOD-SCID mice. However, the limitations of the method render it necessary to search for specific markers based on SP properties, as suggested in our genomic study.

## Supporting Information

Table S1 List of genes up- and down- regulated in epithelial and stromal SP versus complete epithelial and stromal cell fractions.
(0.46 MB DOC)

Table S2 Top ten up- and down-regulated genes in both, epithelial and stromal SP sorted population.
(0.06 MB DOC)

Table S3 Common gene signature of the human endometrial epithelial and stromal SP.
(0.10 MB DOC)
