# Supplementary material for: Human Endometrial Side Population Cells Exhibit Genotypic, Phenotypic and Functional Features of Somatic Stem Cells
Source: PLoS One. 2010 Jun 24;5(6):e10964. doi: 10.1371/journal.pone.0010964 (PMC2891991; doi:10.1371/journal.pone.0010964)
Supplement: Table S1 — List of genes up- and down- regulated in epithelial and stromal SP versus complete epithelial and stromal cell fractions. (0.46 MB DOC) [file pone.0010964.s001.doc]

**TABLE S1.** List of genes up and down regulated in epithelial and stromal SP cells versus whole epithelial and stromal cell fractions.

196 up and 117 down regulated genes in epithelium.

121 up and 73 down regulated genes in stroma.

| FC(SP_Epit/Epit) | GeneName |
| --- | --- |
| 19,822537 | IL1B |
| 19,0688974 | CXCL1 |
| 16,887021 | HSPA6 |
| 13,6609517 | TUBA1 |
| 13,4015637 | CCL4 |
| 13,3207524 | POLR2J2 |
| 13,2809492 | CACNG5 |
| 12,8529431 | BC015216 |
| 12,4871459 | GDF15 |
| 11,9711707 | CD69 |
| 11,6039477 | RGS1 |
| 11,5529517 | CKM |
| 11,4554802 | ASB16 |
| 11,4499142 | CLCN5 |
| 11,3810354 | FOXD2 |
| 11,2599982 | DNM1 |
| 10,7305396 | XCL1 |
| 10,4565283 | AK126019 |
| 10,2128075 | PPP1R2 |
| 10,1881271 | ZNF2 |
| 10,1072702 | CCDC93 |
| 10,0931983 | HS6ST3 |
| 9,95074115 | XCL2 |
| 9,81530766 | SAMSN1 |
| 9,56728919 | KLRB1 |
| 9,52750078 | C1orf187 |
| 9,4153014 | ADM |
| 9,30663952 | SLC2A14 |
| 9,24211923 | CDKAL1 |
| 9,21832401 | TAGAP |
| 9,02139745 | ENST00000321892 |
| 8,90184696 | CCL3 |
| 8,8704455 | NR4A3 |
| 8,6709333 | ENST00000358378 |
| 8,63954855 | AREG |
| 8,61713475 | KRT86 |
| 8,52762287 | DKK2 |
| 8,52182346 | TNFAIP3 |
| 8,48479698 | ACBD7 |
| 8,29915216 | PHLDA2 |
| 8,28568068 | ZNF702 |
| 8,28335363 | THC2585201 |
| 8,24930156 | HNRPLL |
| 8,23197734 | SLC27A1 |
| 8,17710888 | LOC57400 |
| 8,17620466 | MMP1 |
| 8,12474704 | RAC2 |
| 8,08194066 | CXCL2 |
| 7,93599359 | PSCDBP |
| 7,92268527 | INHBA |
| 7,84151036 | TMEM119 |
| 7,83656177 | R3HDM2 |
| 7,80270653 | ZFHX1B |
| 7,7992012 | BLZF1 |
| 7,77891934 | GZMA |
| 7,77217024 | ZCCHC2 |
| 7,76919661 | ZNF683 |
| 7,76275113 | LOC222967 |
| 7,72410445 | PSPH |
| 7,35447931 | TRBV5-4 |
| 7,28280946 | CCDC16 |
| 7,2794745 | SERPINB2 |
| 7,11698116 | SLC7A11 |
| 6,9802191 | CCL5 |
| 6,94094569 | ZNF713 |
| 6,93417993 | AIF1 |
| 6,89559517 | OR2C3 |
| 6,8955429 | IER5 |
| 6,8735609 | HNRPA3 |
| 6,85396241 | C6orf128 |
| 6,84662449 | PPID |
| 6,82063674 | AGGF1 |
| 6,80831902 | CD300A |
| 6,78726434 | CD96 |
| 6,70839719 | AL833655 |
| 6,68769793 | ANGPTL4 |
| 6,66861366 | FAM40B |
| 6,65532312 | CD83 |
| 6,65098627 | AY358219 |
| 6,61664211 | DGKE |
| 6,56898513 | MGC14436 |
| 6,50061265 | GLMN |
| 6,49434097 | PRG1 |
| 6,44958719 | THC2681759 |
| 6,44681328 | RAC2 |
| 6,34414362 | RND3 |
| 6,29924129 | OLIG1 |
| 6,22752276 | C10orf4 |
| 6,197737 | IL8 |
| 6,19654237 | AD7C-NTP |
| 6,17389599 | FAM19A4 |
| 6,16629348 | LTB |
| 6,12257123 | GPR68 |
| 6,09869222 | GBP6 |
| 6,06717193 | TDH |
| 5,96911881 | HES1 |
| 5,93587832 | C19orf16 |
| 5,91432706 | IER3 |
| 5,85501989 | IL2RB |
| 5,83331754 | BCL2A1 |
| 5,79502118 | GZF1 |
| 5,7820695 | PTGER4 |
| 5,77383619 | HLA-DPB1 |
| 5,73424466 | AK022155 |
| 5,69706144 | FXYD5 |
| 5,69575006 | RGS2 |
| 5,62936193 | CSF2 |
| 5,61060554 | MMP3 |
| 5,59447446 | C8orf46 |
| 5,53557695 | TBX21 |
| 5,50303335 | COTL1 |
| 5,43950688 | TAGAP |
| 5,42115762 | S81524 |
| 5,38039291 | THC2666580 |
| 5,37851127 | TRPV2 |
| 5,34295703 | ASPA |
| 5,29615025 | HES1 |
| 5,25796277 | PLK3 |
| 5,24780389 | CXCL10 |
| 5,22530803 | ALOX5AP |
| 5,19498762 | BBC3 |
| 5,19117296 | AF131795 |
| 5,19114972 | SLC2A3 |
| 5,18137882 | CLEC2B |
| 5,17911219 | PCDHB6 |
| 5,15776229 | ARHGAP9 |
| 5,13789086 | LAT2 |
| 5,13310338 | HCST |
| 5,11402743 | XKR6 |
| 5,07445215 | AF312387 |
| 5,03650214 | SLA |
| 4,9694205 | EGR2 |
| 4,93243236 | PMAIP1 |
| 4,90740863 | SOD2 |
| 4,90275248 | ITGAX |
| 4,88283265 | EGR3 |
| 4,84970192 | TUBB3 |
| 4,79845693 | GEM |
| 4,79418451 | IFNG |
| 4,73789147 | IL4I1 |
| 4,73739249 | THC2609820 |
| 4,72988727 | CCL3L3 |
| 4,72368523 | TRAF3IP3 |
| 4,70469467 | CD86 |
| 4,69739303 | CYFIP2 |
| 4,68163387 | CD48 |
| 4,67993926 | RGR |
| 4,65828282 | HCLS1 |
| 4,62771737 | TMEFF2 |
| 4,6247017 | TAF1A |
| 4,62227299 | IRX5 |
| 4,60205057 | FLJ39575 |
| 4,59979969 | TGFBI |
| 4,59173594 | FLJ21438 |
| 4,58926906 | DOCK8 |
| 4,55763806 | RRAD |
| 4,53263952 | SPOCK2 |
| 4,51163439 | G0S2 |
| 4,49797285 | IL6 |
| 4,4968673 | SYNE1 |
| 4,46540171 | COTL1 |
| 4,46407768 | CD247 |
| 4,45459801 | GZMH |
| 4,44386215 | HSPA1A |
| 4,41569327 | CYR61 |
| 4,34514095 | KLRC1 |
| 4,31419755 | IL23A |
| 4,29324783 | TYROBP |
| 4,28650406 | GBP5 |
| 4,26839226 | ZAP70 |
| 4,26732273 | IL18RAP |
| 4,2566798 | CCL3L3 |
| 4,24168474 | U94903 |
| 4,23934089 | SERTAD1 |
| 4,23021342 | ICAM1 |
| 4,22878273 | TNFRSF19L |
| 4,21492934 | ARHGDIB |
| 4,17821055 | THC2541992 |
| 4,17129745 | GZMB |
| 4,11520457 | PDE4B |
| 4,09018842 | TNF |
| 4,0467632 | JAK3 |
| 4,04171637 | HSPA1A |
| 4,01347019 | FGR |
| 4,01029352 | BCAS4 |
| 3,99858366 | PLK2 |
| 3,95783578 | LRMP |
| 3,92540961 | RELB |
| 3,92416665 | SH2D1B |
| 3,91888694 | PTPRC |
| 3,86420587 | LRRC2 |
| 3,85437709 | LCP1 |
| 3,76825681 | DUSP5 |
| 3,64774067 | GADD45A |
| 3,56397398 | CXCL2 |
| 3,55645943 | EBI2 |
| -3,0991039 | VWF |
| -3,7794121 | PFDN6 |
| -3,809332 | ALAD |
| -3,8324792 | TPX2 |
| -3,850505 | NPBWR1 |
| -3,8515813 | PRKAB1 |
| -3,8746484 | ZFYVE9 |
| -3,8886333 | CYP2J2 |
| -3,9050599 | RNF125 |
| -3,9156835 | GJB1 |
| -3,9289892 | KIAA1919 |
| -3,9318519 | ARHGAP26 |
| -3,9408552 | LOC649828 |
| -3,9474401 | EPB41L4B |
| -3,9745229 | ARL4D |
| -3,9824713 | EEF2 |
| -3,9956826 | MGC46336 |
| -4,0028983 | PDAP1 |
| -4,0139662 | ZNF552 |
| -4,0156238 | AKAP13 |
| -4,0304746 | BC014938 |
| -4,03623 | SREBF2 |
| -4,0477039 | MAPK13 |
| -4,0499155 | TMEM28 |
| -4,0534498 | CYB561 |
| -4,055571 | TAF5L |
| -4,0627061 | X03757 |
| -4,0767417 | SMARCD2 |
| -4,0808483 | TMEM101 |
| -4,0813939 | YAP1 |
| -4,0999502 | BRD3 |
| -4,1089332 | AK056260 |
| -4,1127977 | DDR1 |
| -4,1288914 | ALPL |
| -4,1428821 | PRPF19 |
| -4,1505713 | THC2624264 |
| -4,2404216 | ST3GAL4 |
| -4,2996529 | SLC5A9 |
| -4,3872673 | DLG2 |
| -4,4444639 | SYNGR2 |
| -4,477447 | HSMPP8 |
| -4,4924026 | LOC393078 |
| -4,4937864 | GJB1 |
| -4,5023421 | HIST1H1A |
| -4,5335133 | NKX1-2 |
| -4,5457911 | ADAMTS8 |
| -4,5502223 | AK123704 |
| -4,5766763 | ATP1A3 |
| -4,6945115 | ATP1A4 |
| -4,714775 | KCNK13 |
| -4,7822066 | MYCN |
| -4,8042396 | CARD9 |
| -4,8228888 | CYP4F2 |
| -4,9659017 | HAGHL |
| -4,9922618 | THC2598362 |
| -5,034194 | NP389850 |
| -5,0388667 | PSORS1C2 |
| -5,0448238 | HBD |
| -5,0899717 | TRAP1 |
| -5,0925153 | AK095945 |
| -5,1041639 | LOC728864 |
| -5,1368103 | AK095583 |
| -5,208298 | HSD17B1 |
| -5,2169504 | SPPL2B |
| -5,2193381 | HBA2 |
| -5,3817595 | RHBDL1 |
| -5,3943381 | PCSK6 |
| -5,5124101 | LOC654048 |
| -5,5345201 | SLC39A7 |
| -5,5524124 | HOXB6 |
| -5,5975208 | ANKDD1A |
| -5,6299023 | UCN2 |
| -5,6411902 | ZNF206 |
| -5,6421279 | PIP5K1C |
| -5,6967622 | CCDC24 |
| -5,7057016 | FOXC1 |
| -5,7059666 | C19orf31 |
| -5,7073886 | LOC731997 |
| -5,7116631 | SP5 |
| -5,7214739 | PFKL |
| -5,7490306 | TRIM35 |
| -5,763141 | THC2678411 |
| -5,8059263 | MOP-1 |
| -5,8123857 | SOX8 |
| -5,8210529 | TNXB |
| -5,82139 | DNAJC4 |
| -5,8401473 | C3AR1 |
| -5,8663258 | APOL6 |
| -5,8744517 | FRMD4A |
| -6,0780339 | THC2674530 |
| -6,0792598 | LOC145694 |
| -6,2454222 | CYP4F2 |
| -6,4187708 | THC2689192 |
| -6,4253149 | BRD3 |
| -6,4882153 | HBB |
| -6,8769546 | LOC650392 |
| -6,8999828 | GPR78 |
| -7,0024086 | SYN1 |
| -7,3149788 | THC2503530 |
| -7,3198107 | LOC146325 |
| -7,5777493 | FLJ35934 |
| -7,5926354 | NEUROG3 |
| -7,6538786 | IER5 |
| -7,654093 | Y10152 |
| -7,6995717 | CMIP |
| -7,7936139 | CACNA1E |
| -7,9686315 | SYNPO |
| -8,0191946 | AK092421 |
| -8,0583567 | THC2559929 |
| -8,5058749 | ACBD4 |
| -8,5308999 | NEUROG1 |
| -8,6885186 | THC2481836 |
| -9,1263482 | POU3F3 |
| -9,2627173 | ZNF575 |
| -11,151046 | PCSK1N |
| -12,401502 | FOXE1 |
| FC(SP_Str/Str) | GeneName |
| 6,531956616 | MMP3 |
| 6,412132673 | RND3 |
| 6,274984001 | SERPINB2 |
| 6,026395913 | SLC4A1 |
| 5,820092928 | ANGPTL4 |
| 5,673514303 | INHBA |
| 5,577558475 | IER3 |
| 5,501627522 | KRT34 |
| 5,367438514 | GDF15 |
| 5,324082955 | ADM |
| 5,189554869 | SOD2 |
| 4,963599654 | IL6 |
| 4,548695719 | CDKN1A |
| 4,498979679 | IL1B |
| 4,469767506 | IL8 |
| 4,436063873 | SERPINE1 |
| 4,330320747 | CXCL1 |
| 4,125849288 | HSPA6 |
| 4,103071599 | STC2 |
| 4,086077119 | GEM |
| 3,865426174 | ELL2 |
| 3,863623365 | FLJ39575 |
| 3,850358591 | CSF2 |
| 3,800036852 | NR4A3 |
| 3,625342539 | DUSP5 |
| 3,423472751 | PTX3 |
| 3,411564405 | MAFF |
| 3,361473727 | ARC |
| 3,293198191 | HBEGF |
| 3,286040798 | SPHK1 |
| 3,227815785 | PHLDA2 |
| 3,204141032 | TNFRSF12A |
| 3,198745692 | LOC338620 |
| 3,142878975 | PBEF1 |
| 3,137454012 | CYR61 |
| 3,084347245 | HBEGF |
| 3,048279198 | CYR61 |
| 2,956054122 | RRAD |
| 2,946010782 | EGR2 |
| 2,936493362 | CXCL2 |
| 2,913687676 | BAG3 |
| 2,87550957 | ICAM1 |
| 2,875481562 | MGC14376 |
| 2,784687517 | PBEF1 |
| 2,766978733 | PBEF1 |
| 2,765965697 | UPP1 |
| 2,682608708 | GADD45A |
| 2,669301674 | HSPH1 |
| 2,663162114 | PPP1R15A |
| 2,655736356 | CDKN1A |
| 2,644335949 | ATF3 |
| 2,631971059 | SERTAD1 |
| 2,624787381 | C15orf48 |
| 2,62117883 | BCL6 |
| 2,604809031 | THBS1 |
| 2,55906312 | CYR61 |
| 2,522922493 | ATF3 |
| 2,506477875 | CXCL2 |
| 2,493452421 | MAP1LC3B |
| 2,410663831 | TRIB1 |
| 2,338753168 | DNAJB1 |
| 2,333603687 | EGR3 |
| 2,33332764 | ZC3H12A |
| 2,322691151 | AXUD1 |
| 2,311810969 | PLK3 |
| 2,272032312 | TUFT1 |
| 2,206979209 | INSIG1 |
| 2,178469069 | SNF1LK |
| 2,147242709 | SLC2A14 |
| 2,14320064 | DNAJB4 |
| 2,1133257 | IFRD1 |
| 2,112376187 | SLC2A3 |
| 2,102965308 | ING1 |
| 2,063196189 | RRAD |
| 2,060018553 | KLF6 |
| 2,024469652 | MAP3K8 |
| 2,013889328 | TNFAIP3 |
| 2,010888597 | LOC284454 |
| 2,004890192 | FEM1C |
| 1,982836719 | IL23A |
| 1,980627027 | TUBA1 |
| 1,952634019 | ANXA1 |
| 1,923376499 | RGS16 |
| 1,920917744 | NP |
| 1,915781235 | THC2717907 |
| 1,90934694 | AX721087 |
| 1,899161431 | ERN1 |
| 1,897845106 | TNFAIP3 |
| 1,887706605 | BEX2 |
| 1,879314731 | NUAK2 |
| 1,878251672 | NR4A1 |
| 1,802312946 | EGR4 |
| 1,801145846 | IRF1 |
| 1,800780933 | BHLHB2 |
| 1,797898933 | HSPA1A |
| 1,786009491 | IL7R |
| 1,761336104 | AREG |
| 1,745645269 | DNAJA1 |
| 1,727368634 | OR2C3 |
| 1,714514228 | FOXC1 |
| 1,706066745 | PMAIP1 |
| 1,691394542 | BCL2A1 |
| 1,690385473 | STK17A |
| 1,662170841 | DEDD2 |
| 1,656683874 | SLC38A1 |
| 1,634048907 | BAMBI |
| 1,632955721 | HSPA1A |
| 1,574911433 | GATA3 |
| 1,563295087 | ID2 |
| 1,563236362 | HES1 |
| 1,540738012 | HES1 |
| 1,513382449 | DUSP2 |
| 1,497852983 | SNF1LK |
| 1,493214288 | SNAI1 |
| 1,380940554 | SLPI |
| 1,360149017 | LIF |
| 1,341107687 | IL4I1 |
| 1,334458572 | CCL3 |
| 1,316746535 | DUSP2 |
| 1,267182739 | CCL3L3 |
| 1,245784343 | EBI2 |
| -1,202582445 | LOC137107 |
| -1,216180449 | HNRPLL |
| -1,219972485 | HBD |
| -1,263102007 | GEFT |
| -1,283035021 | CD69 |
| -1,301192226 | THC2598362 |
| -1,310304042 | POLR2J2 |
| -1,31802837 | PKIB |
| -1,340906812 | ASPM |
| -1,359132626 | RGS1 |
| -1,391715487 | CXCL14 |
| -1,396817207 | LOC146325 |
| -1,408093476 | LOC730887 |
| -1,480012878 | SP5 |
| -1,484618128 | ATN1 |
| -1,488781364 | TMEM101 |
| -1,505202367 | LOC390413 |
| -1,535689397 | MYCN |
| -1,536142164 | LOC731599 |
| -1,565910159 | CIRBP |
| -1,601905968 | H1F0 |
| -1,612046622 | XCL1 |
| -1,630556807 | KLRB1 |
| -1,662112029 | FRMD4A |
| -1,750228772 | KIFC1 |
| -1,815306063 | AF334588 |
| -1,816814724 | THC2532927 |
| -1,823953542 | H19 |
| -1,844016319 | THC2624264 |
| -1,878708379 | PCSK1N |
| -1,880832806 | LMNB1 |
| -1,895919685 | CIRBP |
| -1,898604467 | HBB |
| -1,989900698 | HSD17B2 |
| -2,038044166 | INE1 |
| -2,0388933 | THC2712697 |
| -2,109701198 | WFDC2 |
| -2,12625595 | C1orf186 |
| -2,152241992 | SLC3A1 |
| -2,260538194 | M27126 |
| -2,270676732 | UGT2B7 |
| -2,284238783 | HLA-DRB5 |
| -2,290036549 | HIST1H1A |
| -2,295557078 | PIGR |
| -2,316443212 | ANK3 |
| -2,361359859 | EYA2 |
| -2,410834445 | WFDC2 |
| -2,438688805 | SLC34A2 |
| -2,567580779 | TLR9 |
| -2,593734543 | EYA2 |
| -2,603131991 | ASRGL1 |
| -2,608998266 | ASRGL1 |
| -2,610944461 | ASRGL1 |
| -2,626138805 | COX11 |
| -2,68008448 | LOC388532 |
| -2,751723781 | AK096677 |
| -2,752910354 | PRR15 |
| -2,759020074 | IGH@ |
| -2,761821218 | HOXB6 |
| -2,833067199 | C20orf85 |
| -2,85208087 | GJB1 |
| -3,082645696 | ST6GALNAC1 |
| -3,085441374 | TPD52L1 |
| -3,105029783 | ACSL5 |
| -3,105081651 | HGD |
| -3,168558192 | SCGB2A1 |
| -3,512853518 | C20orf85 |
| -3,566919298 | SOX17 |
| -3,588907421 | ASRGL1 |
| -3,608975251 | SERPINA5 |
| -4,184575233 | SERPINA5 |
| -4,3210118 | SCGB1D2 |
| -4,331139511 | VWF |
| -4,73484651 | SCGB1D1 |
