# Supplementary material for: Human Endometrial Side Population Cells Exhibit Genotypic, Phenotypic and Functional Features of Somatic Stem Cells
Source: PLoS One. 2010 Jun 24;5(6):e10964. doi: 10.1371/journal.pone.0010964 (PMC2891991; doi:10.1371/journal.pone.0010964)
Supplement: Table S2 — Top ten up- and down-regulated genes in both, epithelial and stromal SP sorted population. (0.06 MB DOC) [file pone.0010964.s002.doc]

**TABLE S2.** Top ten up & down regulated genes in epithelial and stromal SP.

| TOP TEN GENES IN EPITHELIAL SP | | |
| --- | --- | --- |
| Gene | Fold Change | Function |
| IL1-B | 19.823 | Anti-apotosis  Negative regulation of cell proliferation  Positive regulation of mitosis  Positive regulation of vascular endothelial growth factor production |
| CXCL1 | 19.069 | Negative regulation of cell proliferation  Intracellular signalling cascade |
| HSPA6 | 16.887 | Stress response |
| TUBA4A | 13.661 | Protein polymerization  GTPase activity  Structural molecule activity |
| CCL4 | 13.402 | Cell adhesion  Cell- cell signalling  Signal traduction |
| POLR2J2 | 13.321 | Transcription |
| CACGN5 | 13.281 |  |
| GDF15 | 12.487 | Cell-cell signalling  Transforming growth factor beta receptor signalling pathway |
| CD69 | 11.971 | Transmembrane receptor activity |
| RGS1 | 11.604 | Negative regulation of signal transduction |
| FOXE1 | -12.402 | Cell migration  Regulation of transcription, DNA-dependent  Regulation of transcription from RNA polymerase II promoter |
| PCSK1N | -11.151 | Receptor binding |
| POU3F3 | -9.126 | Transcription factor activity |
| NEUROG1 | -8.531 | Regulation of transcription from RNA polymerase II promoter |
| SYNPO | -7.969 | Protein binding |
| CACNA1E | -7.794 | Calcium ion transport |
| CRHR2 | -7.654 | G- protein signalling |
| IER5 * | -7.654 |  |
| NEUROG3 | -7.593 | Regulation of transcription |
| SYN1 | -7.002 | Transporter activity  ATP binding |
| TOP TEN GENES IN STROMAL SP | | |
| Gene | Fold Change | Function |
| MMP3 | 6.532 | Proteolysis  Collagen catabolic process |
| RND3 | 6.412 | Cell adhesion  Small GTPase mediated signal transduction |
| SERPINB2 | 6.275 | Anti-apoptosis |
| SLC4A1 | 6.026 | Cellular ion homeostasis |
| ANGPTL4 | 5.820 | Cell differentiation  Negative regulation of apoptosis  Response to hypoxia |
| INHBA | 5.674 | Induction of apoptosis  Negative regulation of cell cycle  Negative regulation of cell growth  Cell cycle arrest |
| IER3 | 5.578 | Anti-apoptosis |
| KRT34 | 5.502 | Structural molecule activity |
| GDF15 | 5.367 | Cell- cell signalling  Transforming growth factor beta factor signalling pathway |
| ADM | 5.324 | Hormone activity |
| VWF | -4.331 | Cell-substrate adhesion |
| SCGB1D2 | -4.321 | Binding |
| SERPINA5* | -4.185 | Binding |
| ASRGL1* | -3.589 | Asparagine catabolic process via L- aspartate |
| SOX17 | -3.567 | Regulation of transcription, DNA-dependent |
| SCGB2A1 | -3.169 | Binding |
| HGD | -3.105 | Oxidation reduction |
| ACSL5 | -3.105 | Fatty acid metabolic process |
| TPD52L1 | -3.085 | G2/M transition of mitotic cell cycle |
| ST6GALNAC1 | -3.083 | Protein amino acid glycosylation |
