# Supplementary material for: Human Endometrial Side Population Cells Exhibit Genotypic, Phenotypic and Functional Features of Somatic Stem Cells
Source: PLoS One. 2010 Jun 24;5(6):e10964. doi: 10.1371/journal.pone.0010964 (PMC2891991; doi:10.1371/journal.pone.0010964)
Supplement: Table S3 — Common gene signature of the human endometrial epithelial and stromal SP. (0.10 MB DOC) [file pone.0010964.s003.doc]

**TABLE S3.** Common gene signature of epithelial and stromal SPs in human endometrium.

| DOWN-REGULATED GENES | | | |
| --- | --- | --- | --- |
| Gene Description | Gene Symbol | Function | Fold Change |
| Von Willebrand factor | VWF | Cell-substrate adhesion | -3,09 (EPIT)  -4,33 (STR) |
| Gap junction protein, beta 1, 32kDa | GJB1 | Cell-cell signalling  Integral to membrane | -3,91 (EPIT)  -2,85 (STR) |
| Transmembrane protein 101 | TMEM101 | Positive regulation of I-KappaB kinase/NF-KappaB cascade  Integral to membrane  Signal transducer activity | -4,08 (EPIT)  -1,48 (STR) |
|  | THC2624264 |  | -4,15 (EPIT)  -1,84 (STR) |
| Histone cluster 1, H1a | HIST1H1A | Nucleosome assembly  DNA binding | -4,50 (EPIT)  -2,29 (STR) |
| V-myc myelocytomatosis viral related oncogene, neuroblastoma derived (avian) | MYCN | Regulation of transcription from RNA polymerase II promoter  Protein binding | -4,78 (EPIT)  -1,53 (STR) |
|  | THC2598362 |  | -4,99 (EPIT)  -1,30 (STR) |
| Hemoglobin delta | HBD | Oxygen transport | -5,04 (EPIT)  -1,21 (STR) |
| Homeobox B6 | HOXB6 | Regulation of transcription, DNA-dependent | -5,55 (EPIT)  -2,76 (STR) |
| Sp5 transcription factor | SP5 | Regulation of transcription, DNA-dependent | -5,71 (EPIT)  -1,48 (STR) |
| FERM domain containing 4A | FRMD4A | Binding | -5,87 (EPIT)  -1,66 (STR) |
| Hemoglobin beta | HBB | Nitric oxide transport  Oxygen transport  Positive regulation of nitric oxide biosynthetic process | -6,48 (EPIT)  -1,89 (STR) |
|  | LOC146325 |  | -7,31 (EPIT)  -1,39 (STR) |
| Proprotein convertase subtilisin/kexin type 1 inhibitor | PCSK1N | Neuropeptide signalling pathway | -11,15 (EPIT)  -1,87 (STR) |
| UP-REGULATED GENES | | | |
| Gene Description | Gene Symbol | Function | Fold Change |
| Interleukin 1beta | IL1B | Anti-apotosis  Negative regulation of cell proliferation  Positive regulation of mitosis  Positive regulation of vascular endothelial growth factor production | +19,82 (EPIT)  + 4,49 (STR) |
| Chemokine (C-X-C motif) ligand 1 (melanoma growth stimulating activity, alpha) | CXCL1 | Negative regulation of cell proliferation  Intracellular signalling cascade | +19,06 (EPIT)  + 4,33 (STR) |
| Heat shock 70kDa protein 6 (HSP70B') | HSPA6 | Stress response | +16,88 (EPIT)  + 4,12 (STR) |
| Tubulin alpha-1 chain | TUBA1 | Protein polymerization | +13,66 (EPIT)  + 1,98 (STR) |
| Growth differentiation factor 15 | GDF15 | Cell-cell signalling  Transforming growth factor beta receptor signalling pathway | +12,48 (EPIT)  + 5,36 (STR) |
| Adrenomedullin | ADM | Hormone activity | +9,41 (EPIT)  +5,32 (STR) |
| Solute carrier family 2, facilitated glucose transporter member 14 | SLC2A14 | Cell differentiation  Transmembrane transport | +9,30 (EPIT)  +2,14 (STR) |
| Chemokine (C-C motif) ligand 3 | CCL3 | Cell-cell signalling  Cellular calcium ion homeostasis  Cell motion | +8,90 (EPIT)  +1,33 (STR) |
| Nuclear receptor subfamily 4, group A, member 3 | NR4A3 | Transcription regulation | +8,87 (EPIT)  +3,80 (STR) |
| Amphiregulin | AREG | Cell-cell signalling  Cell proliferation  Positive regulation of DNA regulation | +8,63 (EPIT)  +1,76 (STR) |
| Tumor necrosis factor, alpha-induced protein 3 | TNFAIP3 | Anti-apotosis  Negative regulation of I-KappaB kinase/NF-KappaB cascade  Ubiquitin-dependent protein catabolic process | +8,52 (EPIT)  +2,01 (STR) |
| Pleckstrin homology-like domain, family A, member 2 | PHLDA2 | Apoptosis | +8,29 (EPIT)  +3,22 (STR) |
| Chemokine (C-X-C motif) ligand 2 | CXCL2 | Chemotaxis  Immune response | +8,08 (EPIT)  +2,93 (STR) |
| Inhibin, beta A | INHBA | Induction of apoptosis  Negative regulation of cell cycle  G1/S transition of mitotic cell cycle  Negative regulation of cell growth | +7,92 (EPIT)  +5,67 (STR) |
| Serpin peptidase inhibitor, clade B (ovalbumin), member 2 | SERPINB2 | Anti-apoptosis | +7,27 (EPIT)  +6,27 (STR) |
| Olfactory receptor, family 2, subfamily C, member 3 | OR2C3 | G-protein coupled receptor protein signalling pathway | +6,89 (EPIT)  +1,72 (STR) |
| Angiopoietin-like 4 | ANGPTL4 | Negative regulation of apoptosis  Cell differentiation  Response to hypoxia | +6,68 (EPIT)  +5,82 (STR) |
| Rho family GTPase 3 | RND3 | Cell adhesion  Small GTPase mediated signal transduction | +6,34 (EPIT)  +6,41 (STR) |
| Interleukin 8 | IL8 | Negative regulation of cell proliferation  Cell-cell signalling  Cell adhesion | +6,19 (EPIT)  +4,46(STR) |
| Hairy and enhancer of split 1 | HES1 | Transcription regulation | +5,96 (EPIT)  +1,56 (STR) |
| Immediate early response 3 | IER3 | Anti-apoptosis | +5,91 (EPIT)  +5,57 (STR) |
| BCL2-related protein A1 | BCL2A1 | Anti-apoptosis | +5,83 (EPIT)  +1,69 (STR) |
| Colony stimulating factor 2 (granulocyte-macrophage) | CSF2 | Positive regulation of DNA replication  Positive regulation of Tyrosine phosphorylation of Stat5 protein | +5,62 (EPIT)  +3,85 (STR) |
| Matrix metallopeptidase 3 (stromelysin 1, progelatinase) | MMP3 | Proteolysis  Collagen catabolic process | +5,61 (EPIT)  +6,53 (STR) |
| Serine/threonine-protein kinase | PLK3 | Cell cycle  Protein amino acid phosphorylation | +5,25 (EPIT)  +2,31 (STR) |
| Solute carrier family 2 (facilitated glucose transporter), member 3 | SLC2A3 | Transmembrane transport | +5,19 (EPIT)  +2,11 (STR) |
| Early growth response 2 (Krox-20 homolog, Drosophila) | EGR2 | Transcription factor activity | +4,96 (EPIT)  +2,94 (STR) |
| Phorbol-12-myristate-13-acetate-induced protein 1 | PMAIP1 | Caspase activation Induction apoptosis | +4,93 (EPIT)  +1,70 (STR) |
| Superoxide dismutase 2, mitochondrial | SOD2 | Oxygen homeostasis  Regulation of transcription from RNA polymerase II promoter | +4,90 (EPIT)  +5,18 (STR) |
| Early growth response 3 | EGR3 | Regulation of transcription, DNA-dependent | +4,88 (EPIT)  +2,33 (STR) |
| GTP binding protein overexpressed in skeletal muscle | GEM | Cell surface receptor linked signal transduction  Small GTPase mediated signal transduction | +4,79 (EPIT)  +4,08 (STR) |
| Interleukin-4-induced protein 1 | IL4I1 | Oxidation reduction | +4,73 (EPIT)  +1,34 (STR) |
| Chemokine (C-C motif) ligand 3-like 3 | CCL3L3 | Negative regulation of cell proliferation  Chemotaxis | +4,72 (EPIT)  +1,26 (STR) |
|  | FLJ39575 |  | +4,60 (EPIT)  +3,86 (STR) |
| Ras-related associated with diabetes | RRAD | Small GTPase mediated signal transduction | +4,55 (EPIT)  +2,95 (STR) |
| Interleukin 6 (interferon, beta 2) | IL6 | Negative regulation of cell proliferation  Positive regulation of anti-apoptosis  Positive regulation of osteoblast differentiation | +4,49 (EPIT)  +4,96 (STR) |
| Heat shock 70 kDa protein 1 | HSPA1A | Anti-apoptosis | +4,44 (EPIT)  +1,79 (STR) |
| Cysteine-rich, angiogenic inducer, 61 | CYR61 | Cell adhesion  Cell proliferation  Regulation of cell growth | +4,41 (EPIT)  +3,13 (STR) |
| Interleukin 23, alpha subunit p19 | IL23A | Tissue remodelling | +4,31 (EPIT)  +1,98 (STR) |
| SERTA domain containing 1 | SERTAD1 | Regulation of transcription, DNA-dependent  Positive regulation of cell proliferation  Regulation of cyclin-dependent protein kinase activity | +4,23 (EPIT)  +2,63 (STR) |
| Intercellular adhesion molecule 1 | ICAM1 | Transmembrane receptor activity  Cell adhesion | +4,23 (EPIT)  +2,87 (STR) |
| Dual specificity phosphatase 5 | DUSP5 | Protein tyrosine phosphatase activity | +3,76 (EPIT)  +3,62 (STR) |
| Growth arrest and DNA-damage-inducible, alpha | GADD45A | Apoptosis  Cell cycle arrest  DNA repair | +3,64 (EPIT)  +2,68 (STR) |
| G-protein coupled receptor 183 | EBI2 | G-protein coupled receptor protein signalling pathway | +3,55(EPIT) +1,24 (STR) |
